# Precision Oncology in Surgery: Patient Selection for Operable Pancreatic Cancer

**DOI:** 10.1097/SLA.0000000000003143

**Published:** 2018-12-18

**Authors:** Stephan B. Dreyer, Mark Pinese, Nigel B. Jamieson, Christopher J. Scarlett, Emily K. Colvin, Marina Pajic, Amber L. Johns, Jeremy L. Humphris, Jianmin Wu, Mark J. Cowley, Angela Chou, Adnan M. Nagrial, Lorraine Chantrill, Venessa T. Chin, Marc D. Jones, Kim Moran-Jones, Christopher Ross Carter, Euan J. Dickson, Jaswinder S. Samra, Neil D. Merrett, Anthony J. Gill, James G. Kench, Fraser Duthie, David K. Miller, Susanna Cooke, Daniela Aust, Thomas Knösel, Petra Rümmele, Robert Grützmann, Christian Pilarsky, Nam Q. Nguyen, Elizabeth A. Musgrove, Peter J. Bailey, Colin J. McKay, Andrew V. Biankin, David K. Chang

**Affiliations:** ∗ Wolfson Wohl Cancer Research Centre, Institute of Cancer Sciences, University of Glasgow, Scotland, United Kingdom; † West of Scotland Pancreatic Unit, Glasgow Royal Infirmary, Glasgow, United Kingdom; ‡ The Kinghorn Cancer Centre, Darlinghurst and Garvan Institute of Medical Research, Sydney, Australia; § Academic Unit of Surgery, School of Medicine, College of Medical, Veterinary and Life Sciences, University of Glasgow, Glasgow Royal Infirmary, Glasgow, United Kingdom; ¶ School of Environmental and Life Sciences, University of Newcastle, Ourimbah, NSW, Australia; || Department of Anatomical Pathology, St. Vincent's Hospital, Sydney, NSW, Australia; ∗∗ Stratified Medicine Scotland, Queen Elizabeth University Hospital, Glasgow, United Kingdom; †† College of Medicine, Veterinary, and Life Sciences, University of Glasgow, Glasgow, United Kingdom; ‡‡ Department of Surgery, Royal North Shore Hospital, St Leonards, Sydney, NSW, Australia; §§ University of Sydney, Sydney, NSW, Australia; ¶¶ School of Medicine, Western Sydney University, Penrith, NSW, Australia; |||| Department of Surgery, Bankstown Hospital, Sydney, NSW, Australia; ∗∗∗ Cancer Diagnosis and Pathology Group Kolling Institute of Medical Research and Department of Anatomical Pathology, Royal North Shore Hospital, Sydney, NSW, Australia; ††† Northern Clinical School, Faculty of Medicine, University of Sydney, Sydney, Australia; ‡‡‡ Department of Tissue Pathology and Diagnostic Oncology, Royal Prince Alfred Hospital, Sydney, NSW, Australia; §§§ Department of Pathology, Queen Elizabeth University Hospital, Glasgow, United Kingdom; ¶¶¶ Illumina Inc, San Diego, CA; |||||| Institute for Pathology, TU Dresden, Dresden, Germany; ∗∗∗∗ Institute of Pathology, Faculty of Medicine, Ludwig-Maximilians-Universität, Munich, Germany; †††† Department of Pathology, Universitätsklinikum Erlangen, Erlangen, Germany; ‡‡‡‡ Department of Surgery, Universitätsklinikum Erlangen, Erlangen, Germany; §§§§ Department of Gastroenterology, Royal Adelaide Hospital, Adelaide, Australia.

**Keywords:** biomarkers, genomics, pancreatic cancer, personalized medicine

## Abstract

**Objective:**

We aimed to define preoperative clinical and molecular characteristics that would allow better patient selection for operative resection.

**Background:**

Although we use molecular selection methods for systemic targeted therapies, these principles are not applied to surgical oncology. Improving patient selection is of vital importance for the operative treatment of pancreatic cancer (pancreatic ductal adenocarcinoma). Although surgery is the only chance of long-term survival, 80% still succumb to the disease and approximately 30% die within 1 year, often sooner than those that have unresected local disease.

**Method:**

In 3 independent pancreatic ductal adenocarcinoma cohorts (total participants = 1184) the relationship between aberrant expression of prometastatic proteins S100A2 and S100A4 and survival was assessed. A preoperative nomogram based on clinical variables available before surgery and expression of these proteins was constructed and compared to traditional measures, and a postoperative nomogram.

**Results:**

High expression of either S100A2 or S100A4 was independent poor prognostic factors in a training cohort of 518 participants. These results were validated in 2 independent patient cohorts (Glasgow, n = 198; Germany, n = 468). Aberrant biomarker expression stratified the cohorts into 3 distinct prognostic groups. A preoperative nomogram incorporating S100A2 and S100A4 expression predicted survival and nomograms derived using postoperative clinicopathological variables.

**Conclusions:**

Of those patients with a poor preoperative nomogram score, approximately 50% of patients died within a year of resection. Nomograms have the potential to improve selection for surgery and neoadjuvant therapy, avoiding surgery in aggressive disease, and justifying more extensive resections in biologically favorable disease.

Although molecular markers are being used more frequently to select patients for systemic targeted agents, only imaging modalities are used to stage patients and assess suitability for operative resection. Decisions on primary surgery or a neoadjuvant approach are made without biological measures of tumor aggressiveness, or the risk of occult metastatic disease. This is exemplified in pancreatic ductal adenocarcinoma (PDAC), which has overtaken breast cancer to become the third most common cause of cancer death in the USA.^1^ Surgical resection offers the only chance of cure, with chemotherapy adding modest benefit, but surgery can be associated with significant morbidity and mortality risk. Even with complete resection and adjuvant chemotherapy, the 5-year survival rate is only approximately 20%,^2–6^ with approximately 30% succumbing within the first year (mostly due to distant metastatic disease).^7,8^ This high metastatic recurrence rate indicates current staging modalities for PDAC cannot identify patients with occult metastases and aggressive biology. For these patients, surgical resection brings uncertain benefit. Whipple pancreaticoduodenectomy can be associated with significant mortality risk and morbidity that leads to long postoperative recovery periods of 3 to 6 months, which presents significant implications on patients’ quality of life.^9^ Hence, better selection methods are urgently needed.

Prognosis prediction tools such as nomograms have been developed for many cancer types to better inform treatment decisions. The most widely used tool in resectable PDAC is the prognostic nomogram developed at the Memorial Sloan-Kettering Cancer Center (MSKCC).^10–13^ These, however, can only be applied after resection as they include clinicopathological variables only available following assessment of the resected specimen.

Numerous molecular biomarkers with potential clinical utility have been studied in PDAC, but few have been independently validated.^14–16^ Our group and others have demonstrated that aberrant expression of S100A2 and S100A4 calcium-binding proteins, both of which function to accentuate tumor aggressiveness and metastasis, are associated with poor survival in PDAC.^17–19^ Using RNA sequencing and methylation arrays, we recently reported hypomethylation of S100A2 is associated with the prognostic “Squamous” subtype of PDAC.^20^ This poor prognostic subtype is consistently defined in molecular classifications of PDAC (also termed Quasi-Mesenchymal^21^ or Basal^22^) and S100A2 remains a highly significant gene in each classifier.^20–22^

Here, we assess and validate the prognostic value of these 2 molecules in 1184 patients. Aberrant expression of these biomarkers stratify patients with resectable pancreatic cancer into 3 distinct prognostic groups in a training set (n = 518) and form the basis of a biomarker-based preoperative nomogram aimed at identifying those at high risk of early recurrence. This nomogram was validated in 2 further cohorts (n = 198 and 468), and the proof-of-concept feasibility of its preoperative use was assessed using preoperative endoscopic ultrasound fine-needle aspiration (EUS-FNA) biopsies.

## METHODS

### Patients and Tissue Specimens

**FIGURE 1:**
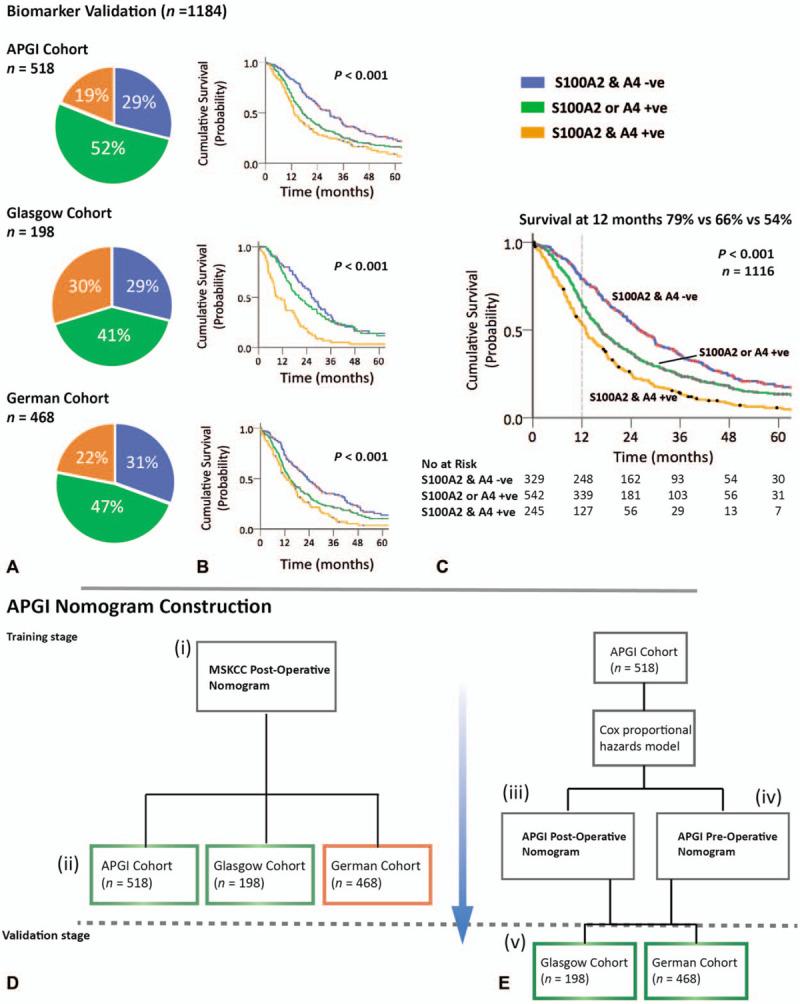
Methodology for biomarker validation and nomogram construction and validation. A, The expression of biomarkers S100A2 and S100A4 was determined using immunohistochemistry in 3 independent cohorts of PDAC and correlated with survival after pancreatectomy. Biomarker expression prevalence is presented as individual pie charts. B and C, Survival following pancreatectomy for all 3 cohorts (b) individually and (c) combined, stratified by biomarker expression (both negative, 1 positive, both positive), is represented by Kaplan-Meier survival curves. Patients with both biomarkers positive had a survival rate of only 54%, 26%, and 6% at 1, 2, and 5 years, respectively. This was found to be 79%, 54%, and 18% in the biomarker negative and 66%, 38%, and 14% in the single biomarker positive groups respectively. D, Clinicopathological variables for all 3 cohorts were independently entered into the MSKCC postoperative nomogram to validate its performance in the patient cohorts. The MSKCC nomogram predicted survival in the APGI (*P* = 5.0 × 10^–5^) and Glasgow cohorts (*P* = 0.025) (green), but not the German cohort (*P* = 0.31) (red). E, The APGI training cohort was used to construct 2 Cox proportional hazard models, 1 was termed the APGI postoperative prognostic nomogram, and 1 the APGI preoperative prognostic nomogram. These were assessed and validated against the Glasgow (*P* = 1.7 × 10^–3^) and German (*P* = 1.2 × 10^–5^) validation cohorts with excellent fit in both cohorts.

Detailed clinicopathological and outcome data were obtained for 3 cohorts of consecutive unselected patients, totaling 1184, with primarily resected PDAC (Fig. 1). None of the patients received neoadjuvant chemo- or radiotherapy. The training cohort of 518 patients were accrued prospectively through the Australian Pancreatic Cancer Genome Initiative (APGI) (www.pancreaticcancer.net.au), for the International Cancer Genome Consortium (ICGC; www.icgc.org).^23^ The 2 independent validation cohorts of 198 and 468 patients were from the West of Scotland Pancreatic unit, Glasgow Royal Infirmary, United Kingdom; and the Technical University of Dresden, Dresden, Germany, respectively (Table 1). Some patients from these cohorts were used for previous studies.^19,20,24–29^ The current study was conducted in accordance with the TRIPOD type 3 model development approach, and REMARK (Reporting Recommendations for Tumor Marker Prognostic Studies) criteria (Supplementary Material).^30^ All patients were treated after 1998 with more modern approaches such as multimodality therapy, and some were part of Phase III randomized-controlled trials such as ESPAC-3.^2^ All cohorts displayed clinical and pathological features consistent with the behavior of PDAC and are similar to published PDAC cohorts worldwide.^31,32^ The diagnosis and all pathological features were reviewed centrally by at least 1 specialist pancreatic histopathologist, and the date and cause of death were obtained from Central Cancer Registries or treating clinicians. RNA sequencing data were generated as part of the APGI's contribution to the ICGC, and sample processing and data analysis was performed as previously described.^20^

**TABLE 1:**
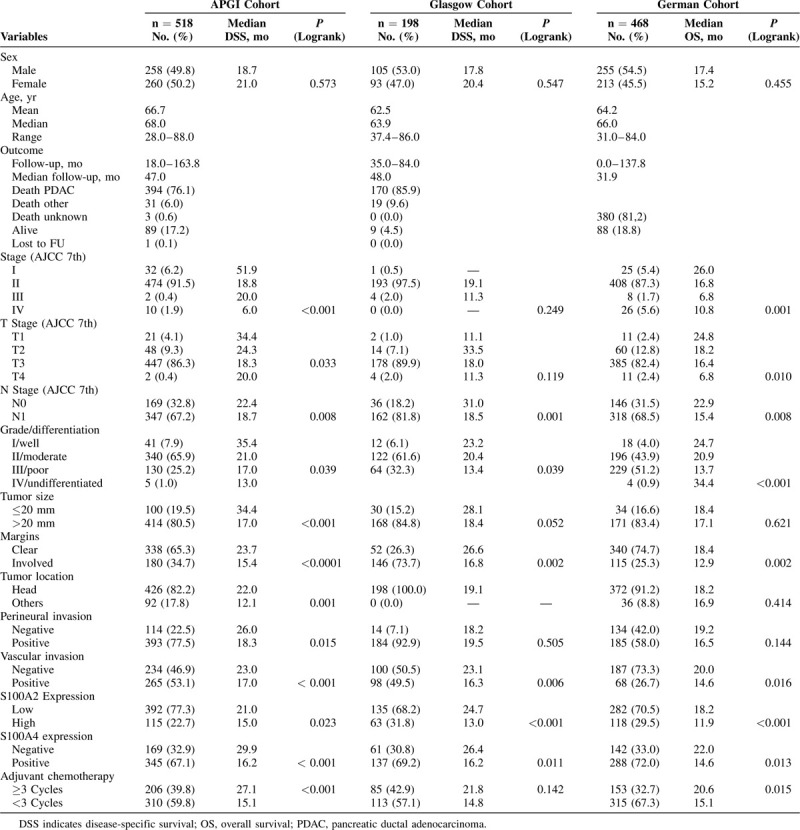
Patient Characteristics for the Australian Pancreatic Cancer Genome Initiative, Glasgow, and the German Cohorts

### Ethical Statement

Ethical approval for the acquisition of data and biological material was obtained from the Human Research Ethics Committee at each participating institution (Supplementary Material).

### Tissue Microarray and Immunohistochemistry (IHC)

Detailed methods for tissue microarray (TMA) construction and immunostaining assessment were described previously.^33^ In brief, immunostaining on resection specimens were performed using TMAs constructed from 3 distinct areas per tumor. TMA sections were incubated with anti-S100A2 mouse monoclonal antibody, 1:50 dilution (clone DAK-S100A2/1; Dako Corporation, Glostrup, Denmark) and anti-S100A4 rabbit polyclonal antibody (NeoMarkers, Cat. No. RB-1804, Fremont, CA) with a dilution of 1:100 for 60 minutes. As part of a standardized biomarker discovery and development process, initial cut-offs were generated using earlier training/discovery cohorts, then validated using independent validation cohorts. S100A2 and S100A4 expression analysis was performed in an early training cohort of PDAC to define the optimal expression for analysis.^19^ High S100A2 expression was defined as cytoplasmic staining with intensity 3+ in >30% of cells and positive S100A4 expression was defined as either nuclear and/or cytoplasmic staining of any intensity in >1% of cells.^19^ Aberrant expression of S100A2 and S100A4 was correlated to survival after pancreatectomy.

### Statistical Analysis

The influence of clinicopathological variables on survival was assessed with Cox proportional hazards regression, and the differences in outcome between predefined subgroups was evaluated using the log-rank test.^34^ Where multiple cohorts were included in a single model, baseline hazard was always stratified by cohort throughout the procedure. On the basis of exploratory analysis, age was modeled with a cohort interaction term in the combined models; no other substantive variable to cohort interactions were identified. *P* values of <0.05 were considered statistically significant. Statistical analysis was performed using SPSS (Version 22.0; IBM SPSS Statistics, IBM Corporation, Armonk, NY). Model fitting and nomogram generation was performed using R 3.4.0 (The R Project for Statistical Computing, Vienna, Austria). Disease-specific survival was used as the primary endpoint for the APGI and Glasgow cohorts. Patients succumbing to other causes were right censored in the analysis. As the majority of patients with PDAC unfortunately succumb to disease, even after seemingly curative resection,^35^ overall survival was used for the German cohort, as disease-specific survival was not available.

### Prognostic Nomograms

#### Memorial Sloan-Kettering Cancer Center Nomogram Evaluation

The published MSKCC nomogram^10^ was applied to the APGI, Glasgow, and German cohorts, yielding per-patient estimates of linear risk score and 6-, 12-, and 24-month survival probabilities (Fig. 1) (Supplementary Material). Some variables in the MSKCC nomogram were not collected in the current cohorts and were imputed to the mean value of that variable reported for the MSKCC nomogram derivation cohort (Table S2).

#### Nomogram Construction

Two Cox proportional hazard models were fit to the APGI training cohort data (Fig. 1), 1 containing conventional clinicopathological variables available postoperatively (age, tumor size, T-stage, tumor location, vascular invasion, perineural invasion, margin status, presence of lymph node metastases, and differentiation), and the other containing only variables assessable preoperatively (age, tumor size, tumor location, and S100A2 and S100A4 status). Patient sex was not included in models due to its known poor prognostic value in PDAC.^10^ To improve the clinical utility of the nomogram, follow-up was truncated at 24 months to focus prognosis prediction in this most clinically critical period after surgery. To simplify generation and application of the predictive nomograms, violations of the proportional hazards assumption were addressed by stratifying the baseline hazard by predictive variables, rather than introducing interaction with a time-dependent stratum.

#### Nomogram Testing

Nomograms were tested for discrimination and calibration against validation cohorts (Glasgow and Germany) using established methods.^36,37^ Variability of the Brier score assessment of overall fit was estimated using 5000 bootstrap rounds.^36,37^

### Endoscopic Ultrasound Fine-needle Aspiration and Cell Block Construction

EUS-FNA samples were collected and processed as per the standard diagnostic pathway using endoscopic and cytohistological techniques according to local practice. Formalin-fixed EUS-FNA tissue fragments or cell block preparations were embedded in paraffin, sectioned (4 μm) and H&E stained as standard. Staining for S100A2 and S100A4 were performed as described above and compared with corresponding resection specimen from 17 consecutive patients undergoing both EUS-FNA and pancreatectomy.

## RESULTS

Patient characteristics are summarized in Table 1, with detailed descriptions provided in the Supplementary Material.

### High S100A2 and S100A4 Expression Is Associated With Poor Survival After Pancreatectomy

**FIGURE 2:**
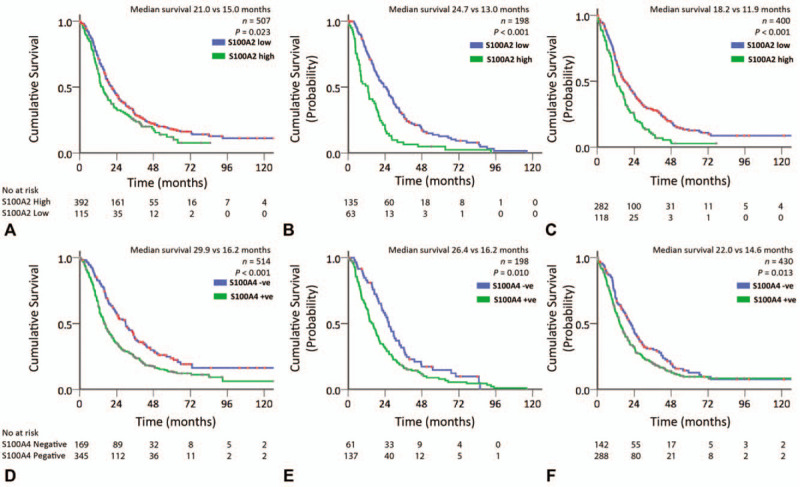
Kaplan-Meier survival curves for S100A2 expression in the (A) APGI, (B) Glasgow, (C) German cohorts; S100A4 expression in (D) APGI, (E) Glasgow, (F) German Cohorts.

In all 3 cohorts, high expression of S100A2 calcium-binding protein was associated with poor survival after pancreatectomy. In the APGI cohort, expression of S100A2 was high in 115 of 507 patients (22.7%) (median survival 21.0 vs 15.0 mo; *P* = 0.023) (Table 1, Fig. 2). S100A2 expression was high in 63 out of 198 patients (31.8%) in the Glasgow cohort (median survival 24.7 vs 13 mo; *P* < 0.001) and in 118 out of 400 patients (29.5%) in the German cohort (median survival 18.2 vs 11.9 mo; *P* < 0.001) (Table 1, Fig. 2).

High S100A2 expression remained a significant independent prognostic factor in a combined multivariate model of all 3 cohorts [Table 2; hazard ratio (HR) = 1.64, 95% confidence interval (CI) 1.33–2.02 *P* < 0.001]. This was also the case in the APGI (Table S3; HR 1.32, 95% CI 0.97–1.80, *P* < 0.001) and Glasgow (Table S4; HR 2.00, 95% CI 1.36–2.90, *P* < 0.001) cohorts, but not the German (Table S5; HR 1.48, 95% CI 0.95–2.29, *P* = 0.076) cohort. It is likely that this reflects reduced power or cohort-specific variable collinearities, as the influence of S100A2 expression on survival was not significantly different between cohorts (Likelihood ratio test, χ^2^ = 4.86, df = 2, *P* = 0.09). Furthermore, S100A2 was associated with poor survival in the German cohort (univariate Cox regression (HR 1.69, 95% CI 1.34 – 2.14, *P <* 0.001) and log rank survival analysis (Fig. 2C). When considered alongside a previous study with more than 400 (200 nonredundant) patients this provides a total of more than 1300 cases supporting the association of high S100A2 expression and poor survival.^19^

**TABLE 2:**
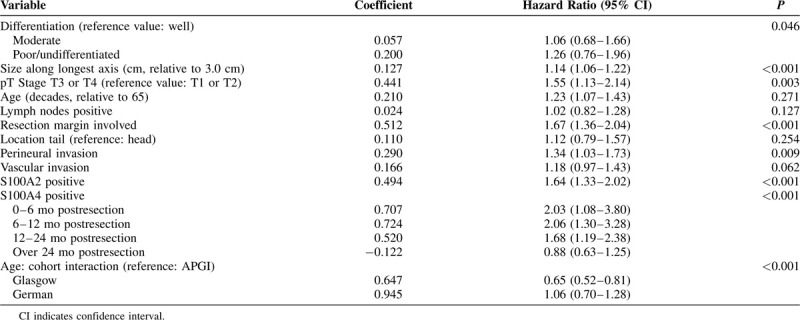
Multivariate Cox Model: All Cohorts Combined (Baseline Hazard Stratified by Cohort)

Positive expression of S100A4 was associated with poor survival after pancreatectomy in all 3 cohorts. In the APGI cohort, 345 out of 514 patients (67.1%) had positive expression of S100A4, which was associated with a significantly worse outcome (median survival 29.9 vs 16.2 mo; *P* < 0.001) (Table 1, Fig. 2). These findings were recapitulated in the Glasgow cohort with 137 out of 198 patients (69.2%) with analyzable tissue demonstrating positive expression of S100A4 that was again associated with poor outcome (median survival 26.4 vs 16.2 mo; *P* = 0.010) (Table 1, Fig. 2). In the German cohort, 288 out of 430 patients (72.0%) with positive S100A4 expression demonstrated significantly worse outcome (median survival 22.0 vs 14.6 mo, *P* = 0.013) (Table 1, Fig. 2).

In a combined multivariate model, S100A4 remained strongly prognostic in a time-dependent manner with its effect on prognosis decreasing after 24 months in both the combined (HR 2.06, 95% CI 1.30–3.28, *P <* 0.001 at 12 mo), and individual models (Table 2, S3–S5). This data suggest that S100A4 is a strong predictor of disease recurrence in the first 24 months after surgery, with its effect decreasing after this period. S100A4 was an independent prognostic factor in multivariate models in the APGI (Table S3, HR 2.13, 95% CI 1.08–4.17, *P =* 0.018 at 12 mo) and Glasgow (Table S5, HR 2.37, 95% CI 0.97–5.79, *P =* 0.048 at 12 mo) cohorts. Similar to S100A2, S100A4 was not significant in a multivariate model in the German cohort (Table S5), however, was predictive in univariate cox regression (HR 1.33, 95% CI 1.06–1.67, *P =* 0.013) and log rank survival analysis (Fig. 2).

### Expression of S100A2 and S100A4 Stratifies Patients Into 3 Prognostic Groups

The combined prognostic effects of the 2 biomarkers were assessed independently in all 3 cohorts (Fig. 1). In all 3 cohorts, tumors with high S100A2 expression were more likely to be associated with positive S100A4 expression (Table S6 in Supplementary Material, *P* ≤ 0.001). In the APGI cohort, patients with low or no expression of either S100A2 or S100A4 had the best prognosis, followed by patients with either high S100A2 or positive S100A4 expression, and patients with both biomarkers positive had the worst prognosis (median survival 29.8 vs 17.0 vs 13.2 mo, respectively, *P* < 0.001) (Fig. 1). These findings were validated in the Glasgow (median survival 26.5 vs 20.1 vs 9.3 mo; *P* < 0.001) and the German (median survival 22.9 vs 14.3 vs 12.9 mo; *P* < 0.001) cohorts (Fig. 1). When combining all 3 cohorts, patients with both biomarkers positive had a 12-month survival rate of only 54% after pancreatectomy, compared to 79% and 66% in the biomarker negative or single biomarker positive groups respectively (*P* < 0.001) (Fig. 1).

There was no difference in the use of adjuvant chemotherapy between patients who were biomarker negative and those with a single biomarker positive in all 3 cohorts (Table S7 in Supplementary Material). In the APGI cohort, patients with both biomarkers positive had lower adjuvant therapy use rate (Table S7, *P* = 0.025). There were no significant differences in adjuvant therapy use amongst biomarker groups in the Glasgow and German cohorts (Table S7 in Supplementary Material).

### Expression of S100A2 and S100A4 Cosegregates With the Squamous Subtype of Pancreatic Ductal Adenocarcinoma

**FIGURE 3:**
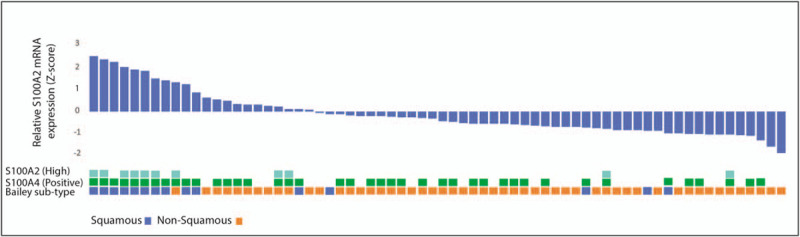
High S100A2 and positive S100A4 expression correlates with the squamous subtype of PDAC. Patients are ranked according to S100A2 mRNA expression and the relative expression *z*-score is represented by a waterfall plot, IHC staining and Bailey subtype is shown below. High S100A2 and positive S100A4 expression associated strongly with the squamous subtype (*P* < 0.001).

In 96 patients who underwent whole transcriptome sequencing analysis as part of the APGI (ICGC) cohort, S100A2 and S100A4 mRNA expression was significantly associated with the recently described squamous subtype^20^ (*P* < 0.01; Fig. 3, Table S8 in Supplementary Material). This was recapitulated in the 235 patients who underwent microarray mRNA expression analysis, as described by Bailey et al.^20^ Using both mRNA and protein expression, patients with high S100A2 (*P* = 0.002) and positive S100A4 (*P* < 0.001) expression were associated with the squamous subtype, with the strongest correlation in those with both biomarkers positive (Fig. 3, Table S9 in Supplementary Material). Squamous subtype tumors^20^ were associated with a significantly higher mean nomogram score than other subtypes (140 vs 103; *P* = 0.004).

### Memorial Sloan-Kettering Cancer Center Prognostic Nomogram Validation for Resectable Pancreatic Ductal Adenocarcinoma

After demonstrating the association of S100A2 and S100A4 expression with the prometastatic squamous subtype and poor prognosis in PDAC, the potential clinical utility of a prognostic nomogram incorporating molecular biomarkers was explored. The overall fit of the published MSKCC nomogram to all 3 cohorts was assessed by stratifying patients using predicted survival score and comparing observed and MSKCC predicted survival [Fig. 1D(i)]. Relative to the true outcome, MSKCC nomogram predictions were optimistic, particularly at later time points, this was especially true for the German cohort (Fig. S1, Supplementary Material). The MSKCC nomogram risk score was prognostic in the APGI and Glasgow cohorts (Cox regression coefficients 0.79 and 1.35, likelihood ratio test for coefficient not zero, *P* = 5.0 × 10^–5^ and 0.025, respectively), but not the German cohort (coefficient 0.15, *P* = 0.31). The MSKCC risk score was well-calibrated against the APGI and Glasgow cohorts (Likelihood-ratio test for coefficient not unity, *P* = 0.28 and 0.56, respectively), but less well against the German cohort (*P* = 2.6 × 10^–9^) [Figs. 1D(ii) and S1, Supplementary Material].

### Preoperative Molecular Nomogram Predicts Survival After Pancreatectomy as Accurately as Postoperative Clinicopathological Nomogram

A molecular nomogram incorporating S100A2 and S100A4 expression was then constructed. The APGI cohort was used to construct 2 prognostic nomograms based on the Cox proportional hazards model: 1 employing traditional postoperatively available variables (“Post-operative Prognostic Nomogram”) [Figs. 1E(iii) and S2, Supplementary Material], and 1 employing only variables that can be measured preoperatively (“Preoperative Prognostic Nomogram”) [Figs. 1E(iv) and 4). To improve the clinical utility of predicting early recurrence and to incorporate the prognostic value of S100A4 over the initial 24 months after surgery, follow-up was truncated at 24 months. Both models included tumor location (pancreatic head vs body/tail), after exploratory analysis in the APGI cohort indicated differences in baseline hazard between these patient groups (Table S10, Supplementary Material).^38^

**FIGURE 4:**
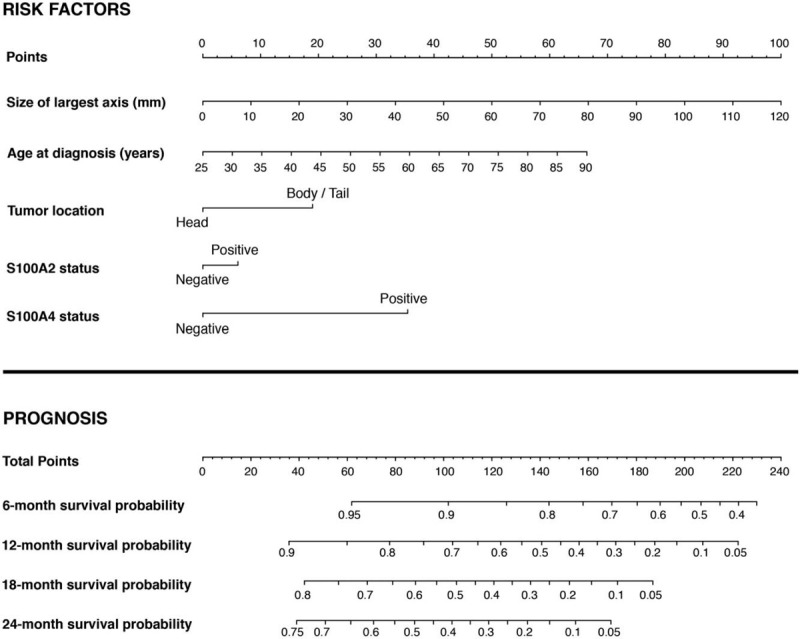
A preoperative molecular prognostic nomogram for resectable pancreatic cancer.

Risk stratification accuracy of the APGI preoperative and APGI postoperative nomograms were assessed in the Glasgow and German validation cohorts. The preoperative prognostic nomogram displayed good discrimination on both validation cohorts (risk score Cox coefficients 0.59 and 0.66, *P* = 1.7 × 10^–3^ and 1.2 × 10^–5^ for Glasgow and German, respectively) [Fig. 1E(v)]. Notably, the APGI preoperative nomogram was superior to the MSKCC postoperative prognostic nomogram in both its spread of risk scores, and the accuracy of its absolute survival estimates (Figs. S1 and S3, Supplementary Material).

The overall accuracy of the APGI preoperative nomogram in predicting patient survival was assessed and compared to the APGI postoperative nomogram. Although the preoperative nomogram was slightly optimistic by predicting marginally better outcome probabilities than those observed in the Glasgow and German cohorts, it was more accurate than the MSKCC postoperative nomogram (Figs. S1 and S3, Supplementary Material). Brier scores was used to formally evaluate the relative performance of the APGI preoperative and postoperative nomograms with more than 5000 bootstrap draws of each validation cohort. This demonstrated the APGI preoperative nomogram was more accurate than the MSKCC postoperative nomogram, and as accurate as the APGI postoperative nomogram in outcome predictions (Figs. S4 and S5, Supplementary Material).

### Preoperative Assessment of Biomarker Expression in Endoscopic Ultrasound Fine-needle Aspiration Samples

**FIGURE 5:**
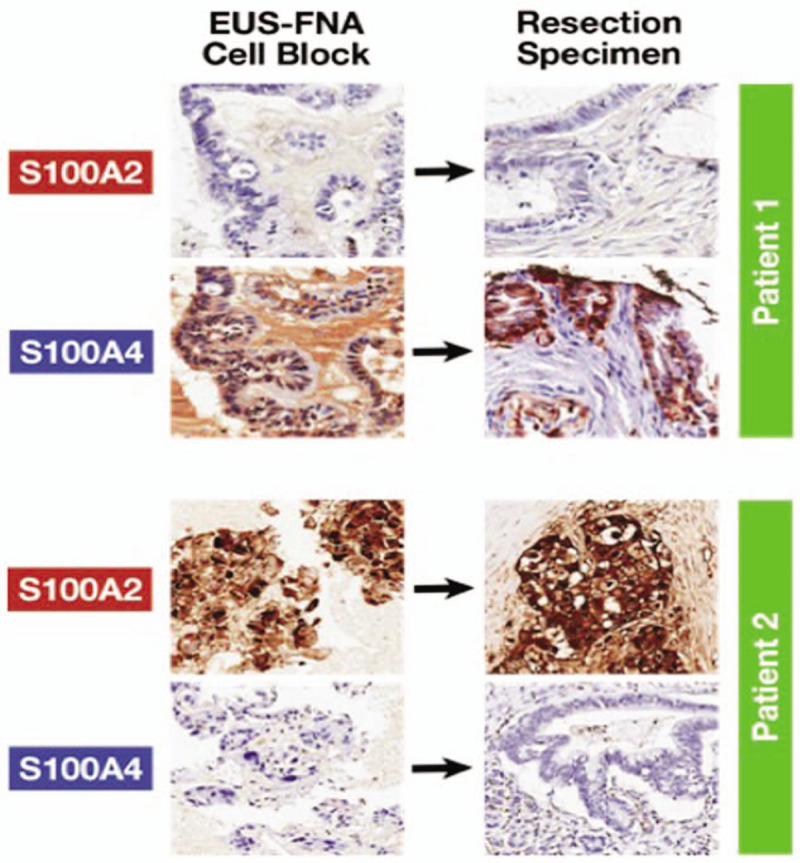
Immunohistochemistry of EUS-FNA versus resection specimen in 2 patients.

A pilot study was performed on 17 consecutive patients to compare biomarker expression status between preoperative EUS-FNA cell blocks and the corresponding surgical resection specimen (Table S11, Supplementary Material). S100A2 and S100A4 expression correlated in 15 (88%) and 14 (82%) out of 17 patients, respectively, based on the EUS-FNA cell block and the surgical specimen for both biomarkers examined (representative images are shown in Fig. 5). This demonstrates that biomarker status can be measured preoperatively using immunohistochemistry, and it is likely that EUS biopsy assessment will improve with the current development of more effective biopsy needles and standardization of processing techniques.

## DISCUSSION

Despite our increasing understanding of the molecular heterogeneity in morphologically identical cancers, and the advances in molecularly guided targeted therapy selection, the impact of these findings in surgical decision-making has not been addressed. Perioperative mortality for pancreatectomy has improved dramatically over the last 30 years and the definition of “resectability” has been expanded over the last decade with increasingly aggressive surgery being performed.^39,40^ Early recurrence, however, remains the Achilles’ heel of surgical resection, making better patient selection for surgery a priority area of research.

In this study, the expression of 2 molecules, S100A2 and S100A4, which functionally promote carcinogenesis and metastasis, were validated as prognostic biomarkers in multiple independent cohorts of patients with resectable PDAC (n = 1184), in keeping with earlier studies (n = ∼400).^19^ These 2 biomarkers were used to stratify patients with resectable PDAC into distinct prognostic phenotypes after pancreatectomy. Patients with both biomarkers positive are at significant risk of early recurrence, with almost half of these patients succumbing within 12 months after pancreatectomy (12-month survival rate = 54%). Suggesting that disease recurrence occur at around 6 months or earlier for the majority of this group. The risk and pattern of disease recurrence following pancreatectomy is not proportional and early recurrence has recently been defined as within 12 months after surgery.^35,41^ We focused on outcome in the first 24 months postoperatively for nomogram construction since the majority of patients that develop early recurrence will succumb to the disease by this point, and thus improve its clinical utility.^41^ A preoperative prognostic nomogram incorporating these 2 biomarkers, and preoperatively determined variables including age, tumor size, and location was developed and independently validated. This preoperative prognostic nomogram performed as well as the published MSKCC postoperative prognostic nomogram, which is the most widely used, and currently considered the criterion standard. In our study, a number of variables used in the MSKCC nomogram were missing and thus comparing the performance of both nomograms in these cohorts is not optimal. The APGI preoperative nomogram, however, uses less variables and all are obtainable before surgical resection to aid decision making. Finally, as a proof-of-concept, biomarker expression status was assessed using immunohistochemical staining of the EUS-FNA cell blocks.

However, due to the retrospective nature of the study, and the cohorts acquired were mature, with long-term follow-up, there are a few limitations with the study. First, a proportion of the recurrence pattern data were not available; therefore, the association between recurrence patterns and biomarker expression was not assessed. Secondly, preoperative CA19-9 measurements were not available for a significant proportion of the patients. Increased serum levels of CA19-9 has been shown to be associated with early recurrence, and may improve the performance of a preoperative nomogram.^41^ Thirdly, only a small number of preoperative EUS samples had sufficient material available for comparison with postoperative S100A2 and S100A4 immunostaining. Therefore, to further validate the clinical utility of the preoperative nomogram, its use should be tested in parallel with trials in PDAC comparing upfront resection and neoadjuvant therapy. Finally, this study was not powered nor designed to assess S100A2 and S100A4 expression and response to adjuvant chemotherapy. Chemotherapy response in the adjuvant setting is difficult to reliably assess as survival difference is dependent on many factors including residual occult metastatic disease and performance status, requiring multicenter personalized medicine trials (such as *PRECISION-Panc* in the UK and *Precision Promise* in the USA) to further delineate this relationship. Interestingly, in the APGI cohort, patients with both biomarkers positive were less likely to be administered adjuvant therapy, possibly due to an aggressive disease phenotype leading to more early recurrence and declining performance status and subsequently reduced adjuvant chemotherapy use.

Aberrant S100A2 and S100A4 expression correlated to the recently described poor prognosis “squamous” (also termed QM or Basal)^21,22^ subtype of PDAC, which is enriched for transcriptional programs associated with proliferation, inflammation, and metastasis.^20^ Mechanistically, S100A2 hypomethylation and associated increased expression is a feature of the squamous subtype. The regulation of S100A4 expression is more complex, potentially involving both tumor and microenvironment factors, and will require further investigation.^42,43^ Interestingly, the squamous subtype was associated with a higher mean nomogram score and demonstrates the potential clinical utility of the currently presented molecular prognostic nomogram in identifying patients with aggressive tumor biology and a prometastatic phenotype. These patients are at high risk of early recurrence and are unlikely to benefit from pancreatectomy and are perhaps better treated with neoadjuvant therapy (an increasingly popular approach to PDAC in many centers), as occult metastatic disease that is not detected by current staging modalities will likely manifest itself during this period. However, the use of neoadjuvant therapy is not universal and a significant proportion of patients may not respond to this approach.^44^ Thus, patients predicted to have a favorable prognosis may be better served with upfront surgery and adjuvant therapy with median survival up to 54 months reported using adjuvant modified FOLFIRINOX in patient cohorts with favorable postoperative prognostic features.^45^ Furthermore, the growing interest in more aggressive and extensive surgery in the setting of borderline resectable or locally advanced disease could be justified by prognostic indicators before initiating therapy.^39,40^ Accurate prognostication can assist multidisciplinary and shared decision making, especially in patients with borderline fitness for surgery, which is a significant proportion of patients with PDAC, delivering a more personalized treatment plan. This approach has the potential to improve the overall outcomes and quality of life for patients with PDAC.

## Supplementary Material

Supplemental Digital Content
