# Supplementary material for: Precision Oncology in Surgery: Patient Selection for Operable Pancreatic Cancer
Source: Ann Surg. 2018 Dec 18;272(2):366–76. doi: 10.1097/SLA.0000000000003143 (PMC7373491; doi:10.1097/SLA.0000000000003143)
Supplement: Supplemental Digital Content [file ansu-272-0366-s001.pdf]

Data Supplement

Supplement to: Precision Oncology in Surgery: Patient Selection for Operable Pancreatic Cancer

Corresponding authors:
David K Chang
Reader and Consultant Surgeon
Institute of Cancer Sciences
Wolfson Wohl Cancer Research Centre
University of Glasgow
Email: David.Chang@glasgow.ac.uk; and

Andrew V Biankin
Regius Professor of Surgery
Institute of Cancer Sciences
Wolfson Wohl Cancer Research Centre
University of Glasgow
Email: Andrew.Biankin@glasgow.ac.uk

Supplementary Methods

Statistical Analysis

The influence of clinicopathological variables on survival was assessed with Cox proportional hazards regression, and the differences in outcome between predefined subgroups was evaluated using the log-rank test.[15] To minimise overfit, the following predetermined protocol was used to fit all explanatory Cox survival models. Data were first pre-processed: continuous variables were median centred, and ordinal factor levels were progressively merged with adjacent values until all levels were supported by at least 30 samples. Limited exploratory analysis was performed, using smoothed Martingale residual plots to establish the likely functional form and possible cohort interaction for continuous predictors. An intermediate model was fit containing all variables as marginal terms, as well as any nonlinear and interaction terms suggested by exploratory analysis. This intermediate model was discarded in favour of a reduced linear-only model if a likelihood ratio test comparing the two resulted in a *P*-value greater than 0.1. The selected model was then tested for violation of the proportional hazards assumption by a global Grambsch-Therneau test, and if a significant deviation was identified ($P < 0.05$), the model was converted to an interval-censored form with time-dependent stratum interactions to restore proportional hazards. This procedure yielded the final explanatory model. Where multiple cohorts were included in a single model, baseline hazard was always stratified by cohort throughout the procedure. On the basis of exploratory analysis, in combined models age was modelled with a cohort interaction term; no other substantive variable to cohort interactions were identified.

Predictive Cox models were fit by a modified version of the above procedure. To focus prediction on the clinically critical period of 24 months following surgery, follow-up was truncated at 24 months. To simplify generation and use of the predictive nomograms, violations of the proportional hazards assumption were addressed by stratifying the baseline hazard by predictive variables, rather than introducing interaction with a time-dependent stratum. All other aspects of model fitting were unchanged from the explanatory model procedure.

P-values of less than 0.05 were considered statistically significant. Statistical analysis was performed using SPSS (Version 22.0; IBM SPSS Statistics, IBM Corporation, Armonk, NY), model fitting and nomogram generation was performed in R 3.4.0 (The R Project for Statistical Computing, Vienna, Austria). Disease-specific survival (DSS) was used as the primary endpoint for the APGI and Glasgow cohorts. Patients succumbing to other causes were right censored in the analysis. As the majority of patients with PC unfortunately succumb to disease, even after seemingly curative resection[16], overall survival (OS) was used for the German cohort, as disease-specific survival was not available.

Ethics approval numbers

APGI:

- Sydney South West Area Health Service Human Research Ethics Committee, Western Zone, protocol number 2006/54
- Sydney Local Health District Human Research Ethics Committee, protocol number X11-0220

- Northern Sydney Central Coast Health Human Research Ethics Committee, protocol number 0612-251M
- Sydney West Area Health Service Human Research Ethics Committee (Westmead Campus), protocol number HREC2002/3/4.19
- South East Sydney Illawarra Area Health, Northern Hospital Network HREC-protocol number 05/321
- South East Sydney Illawarra Area Health HREC- Southern Section, protocol number 05/54

Glasgow:

- West of Scotland Research Ethics Service (WoSRES) committee, NHS Greater Glasgow and Clyde-Molecular profiling of pancreatic cancer for improved prediction of Survival. Research Ethics Committee reference number: 07/S0704/26

Germany:

- Ethikkommission an der Technischen Universität Dresden (Approval number EK59032007) and Ethik-Kommission der FAU (Approval number 170_16 B)

TRIPOD Checklist: Prediction Model Development and Validation				
**Section/Topic**			**Checklist Item**	**Page**
**Title and abstract**				
Title	1	D;V	Identify the study as developing and/or validating a multivariable prediction model, the target population, and the outcome to be predicted.	1
Abstract	2	D;V	Provide a summary of objectives, study design, setting, participants, sample size, predictors, outcome, statistical analysis, results, and conclusions.	5
**Introduction**				
Background and objectives	3a	D;V	Explain the medical context (including whether diagnostic or prognostic) and rationale for developing or validating the multivariable prediction model, including references to existing models.	7, 8
	3b	D;V	Specify the objectives, including whether the study describes the development or validation of the model or both.	8
**Methods**				
Source of data	4a	D;V	Describe the study design or source of data (e.g., randomized trial, cohort, or registry data), separately for the development and validation data sets, if applicable.	9 – 11, Data Supp
	4b	D;V	Specify the key study dates, including start of accrual; end of accrual; and, if applicable, end of follow-up.	Data Supp
Participants	5a	D;V	Specify key elements of the study setting (e.g., primary care, secondary care, general population) including number and location of centres.	Data Supp
	5b	D;V	Describe eligibility criteria for participants.	Data Supp
	5c	D;V	Give details of treatments received, if relevant.	Data Supp
Outcome	6a	D;V	Clearly define the outcome that is predicted by the prediction model, including how and when assessed.	9 - 17
	6b	D;V	Report any actions to blind assessment of the outcome to be predicted.	
Predictors	7a	D;V	Clearly define all predictors used in developing or validating the multivariable prediction model, including how and when they were measured.	Data Supp
	7b	D;V	Report any actions to blind assessment of predictors for the outcome and other predictors.	
Sample size	8	D;V	Explain how the study size was arrived at.	9, Data Supp
Missing data	9	D;V	Describe how missing data were handled (e.g., complete-case analysis, single imputation, multiple imputation) with details of any imputation method.	Data Supp
Statistical analysis methods	10a	D	Describe how predictors were handled in the analyses.	Data Supp
	10b	D	Specify type of model, all model-building procedures (including any predictor selection), and method for internal validation.	9 - 10, Data Supp
	10c	V	For validation, describe how the predictions were calculated.	9 - 10 Data Supp
	10d	D;V	Specify all measures used to assess model performance and, if relevant, to compare multiple models.	9 – 10, Data Supp
	10e	V	Describe any model updating (e.g., recalibration) arising from the validation, if done.	N/A
Risk groups	11	D;V	Provide details on how risk groups were created, if done.	9-10, Data Supp
Development vs. validation	12	V	For validation, identify any differences from the development data in setting, eligibility criteria, outcome, and predictors.	9-10, Data Supp
**Results**				
Participants	13a	D;V	Describe the flow of participants through the study, including the number of participants with and without the outcome and, if applicable, a summary of the follow-up time. A diagram may be helpful.	N/A
	13b	D;V	Describe the characteristics of the participants (basic demographics, clinical features, available predictors), including the number of participants with missing data for predictors and outcome.	Data Supp
	13c	V	For validation, show a comparison with the development data of the distribution of important variables (demographics, predictors and outcome).	Data Supp

Model development	14a	D	Specify the number of participants and outcome events in each analysis.	9-17
	14b	D	If done, report the unadjusted association between each candidate predictor and outcome.	Data Supp
Model specification	15a	D	Present the full prediction model to allow predictions for individuals (i.e., all regression coefficients, and model intercept or baseline survival at a given time point).	Data Supp
	15b	D	Explain how to the use the prediction model.	Data Supp
Model performance	16	D;V	Report performance measures (with CIs) for the prediction model.	11 - 17
Model-updating	17	V	If done, report the results from any model updating (i.e., model specification, model performance).	N/A
**Discussion**				
Limitations	18	D;V	Discuss any limitations of the study (such as nonrepresentative sample, few events per predictor, missing data).	17- 18
Interpretation	19a	V	For validation, discuss the results with reference to performance in the development data, and any other validation data.	17- 18
	19b	D;V	Give an overall interpretation of the results, considering objectives, limitations, results from similar studies, and other relevant evidence.	17- 18
Implications	20	D;V	Discuss the potential clinical use of the model and implications for future research.	17- 18
**Other information**				
Supplementary information	21	D;V	Provide information about the availability of supplementary resources, such as study protocol, Web calculator, and data sets.	N/A
Funding	22	D;V	Give the source of funding and the role of the funders for the present study.	2

Supplementary Results

APGI Cohort

The APGI cohort consisted of 518 patients, most whom were treated after 1998 with more modern therapeutic modalities including adjuvant chemotherapy. There were 260 women and 258 men. The median age at diagnosis was 68 years and range from 28 to 88 years. The median follow-up for surviving patients was 47 months (range, 18 to 164 months). Eighty-nine patients (17.2%) were alive at the census date. Three hundred and ninety-four patients (76.1%) died from pancreatic cancer, thirty-one patients (6%) died of other causes, and three patients (0.6%) died of unknown causes. One patient (0.1%) was lost to follow-up. The median disease-specific survival was 17.9 months, with 3- and 5-year survival rates of 29% and 17% respectively. The majority of tumors were moderately differentiated (Grade II) (66%), followed by poor differentiation (Grade III) (25%), and only 8% of tumors were well differentiated (Grade I). Most tumors were located in the head of the pancreas

(82.2%) and were more than 20 mm in maximal diameter (80.5%). Three hundred and thirty-eight out of 518 patients (65.3%) had resections with clear surgical margins using the R0 = 0 mm definition. Lymph node metastases were present in three hundred and forty-seven (67.2%) patients, perineural invasion was present in three hundred and ninety-three patients (77.5%), and vascular space invasion was present in two hundred and sixty-five patients (53.1%) (Table 1).

Factors associated with a significantly better survival on univariate analysis included T1 and T2 tumors (median survival 31.0 Vs 18.3 months; *P* = 0.006) compared to T3 tumors, well or moderately differentiated tumors (median survival 21.2 vs 17.0 months; *P* = 0.036), absence of lymph node metastases (22.4 Vs 18.7 months; *P* = 0.009), absence of surgical margin involvement (23.7 Vs 15.4 months; *P* < 0.001), tumors of the pancreatic head (median survival 22.0 Vs 12.1 months; *P* < 0.001) compared with those of the body/tail, absence of vascular space invasion (23.0 Vs 17.0 months; *P* < 0.001), and absence of perineural invasion(median survival 26.0 vs 18.3 months; *P = 0.016*).

Glasgow Cohort

The Glasgow cohort consisted of 198 patients, which included 93 women and 105 men. The mean age at diagnosis was 63 years and range from 37 to 86 years. The median follow-up for surviving patients was 48 months (range, 35 to 84 months). Nine patients (4.5%) were alive at the census date. One hundred and seventy patients (85.9%) died from pancreatic cancer, nineteen patients (9.6%) died of other causes, and no patients were lost to follow-up. The median disease-specific survival was 17.0 months, with 3- and 5-year survival rates of 22% and 10% respectively.

The majority of tumors were moderately differentiated (Grade II) (62%), followed by poor differentiation (Grade III) (32%), and only 6% of tumors were well differentiated (Grade I). All tumors were located in the head of the pancreas and most were more than 20 mm in maximal diameter (84.8%). 52 out of 198 patients (26.3%) had resections with clear surgical margins using the R0 = 1 mm definition. Lymph node metastases were present in one hundred and sixty-two (81.8%) patients, perineural invasion was present in one hundred and eighty-four patients (92.9%), and vascular space invasion was present in ninety-eight patients (49.5%) (Table 1).

Factors associated with a significantly better survival on univariate analysis included female sex (median survival 20.4 vs 17.0 months, P = 0.036), well and moderately differentiated tumors (median survival 20.9 vs 13.4 months, P = 0.016), T1 and T2 tumors (median survival 33.5 Vs 17.8 months; P = 0.038) compared to T3 tumors, absence of lymph node metastases (31.0 Vs 18.8 months; P = 0.001), absence of surgical margin involvement (26.6 Vs 16.8 months; P = 0.002), and absence of vascular space invasion (23.1 Vs 16.3 months; P = 0.006).

German Cohort

The German cohort consisted of 468 patients that were treated at three units (Universities of Dresden, Regensburg and Jena), which included 213 women and 255 men. The mean age at diagnosis was 64 years and range from 31 to 84 years. The median follow-up for surviving patients was 31.9 months (range, 0 to 137 months). Eighty-eight patients (18.8%) were alive at the census date. Accurate disease specific survival was not available for this cohort. The median overall survival was 15.7 months, with 3- and 5-year survival rates of 25% and 11% respectively. The majority of tumors were poorly differentiated (Grade III) (51%),

followed by moderate differentiation (Grade II) (44%), and only 4% of tumors were well differentiated (Grade I). Three hundred and seventy-two tumors (91.2%) were located in the head of the pancreas and for those with accurate size documented, most were more than 20 mm in maximal diameter (83.4%). 340 out of 486 patients (74.7%) had documented resections with clear surgical margins using the R0 = 0 mm definition. Lymph node metastases were present in three hundred and eighteen (68.5%) patients, perineural invasion was documented positive in one hundred and eighty-five patients (58.0%), and vascular space invasion was documented as present in sixty-eight patients (26.7%) (Table 1).

Factors associated with a significantly better survival on univariate analysis included well to moderately differentiated tumors (median survival 21.1 vs 13.8 months; $P < 0.001$), absence of lymph node metastases (median survival 22.9 vs 15.4 months; $P = 0.008$), absence of surgical margin involvement (median survival 18.4 vs 12.9 months; $P = 0.002$) and absence of vascular space invasion (median survival 20.0 vs 14.6 months; $P = 0.016$) (Table 1).

Table S1: REMARK summary for S100A2 and A4

Category	Summary
**Introduction**	
**Markers Examined**	S100A2 (S100 calcium binding protein A2)<br>S100A4 (S100 calcium binding protein A4)
**Objective**	Assess the potential of S100A2 and S100A4 expression as prognostic biomarkers in patients with resectable pancreatic adenocarcinoma
**Hypothesis**	S100A2 and A4 expression co-segregates patients with differential outcomes in pancreatic ductal adenocarcinoma
**Patients & Methods**	
**Patients**	518 (APGI original cohort), 198 (Glasgow validation cohort) and 468 (German validation cohort) patients who underwent pancreatic resection for pancreatic ductal adenocarcinoma with curative intent (Table 1; data supplement)
**Specimen characteristics**	TMAs constructed from formalin-fixed, paraffin-embedded surgical specimens, each patient represented by 3 x 1mm cores
**Assay methods**	Immunohistochemistry performed on TMAs, which were score by two independent assessors blinded to outcomes, of whom at least 1 is a specialist pancreatic pathologist
**Study design**	Retrospective analysis of prospectively maintained database of cohorts of consecutive patients associated with Australian Pancreatic Genome Initiative (Sydney, Australia) for APGI cohort; West of Scotland Pancreatic Unit, Glasgow Royal Infirmary (Glasgow, United Kingdom) for Glasgow validation cohort; and University of Dresden (Dresden, Germany) for the German validation cohort<br>End points were cancer specific survival for the APGI and Glasgow cohorts, overall survival for the German cohort<br>Clinicopathological features summarised in Table 1 and data supplement
**Statistical analysis methods**	Median survival estimated using the Kaplan-Meier method; difference tested using log rank test<br>Clinicopathological variables analysed with $P < 0.10$ on log-rank test were entered into Cox proportional hazards multivariate analysis; models generated using backward elimination of redundant variables<br>Patients were dichotomised into high / positive and low / negative:<br>S100A2 expression groups based on cytoplasmic intensity in > 30% of cells<br>S100A4 expression groups based on any nuclear or cytoplasmic staining<br>There were some missing biomarker data for small numbers of patients due to loss of cores on TMAs during processing, these include for APGI cohort 11 for S100A2 and 4 for S100A4, for German validation cohort 68 for S100A2 and 38 for S100A4. No biomarker results were missing for the Glasgow cohort
**Results**	
**Data analysis and presentation**	Clinicopathological characteristics are comprehensively described in Table 1 and the data supplement<br>S100A2 expression associated with a worse prognosis following pancreatectomy in a combined multivariate model of all 3 cohorts (Table 2; HR = 1.64, 95% CI 1.33 – 2.02 $P < 0.001$); the APGI (21.0 vs 15.0 months, $P = 0.023$; HR 1.32, 95% CI 0.97 – 1.80, $P < 0.001$) and Glasgow cohorts (24.7 vs 13.0 months, $P < 0.001$; HR 2.00, 95% CI 1.36 – 2.90, $P < 0.001$), but not the German (18.2 vs 11.9 months, $P < 0.001$; HR 1.48, 95% CI 0.95 – 2.29, $P = 0.076$) cohort.<br>S100A4 expression associated with a worse prognosis following pancreatectomy in a time dependent manner with its effect on prognosis decreasing after 24 months in the combined (HR 2.06, 95% CI 1.30 – 3.28, $P < 0.001$ at 12 months), the APGI (29.9 vs 16.2 months, $P < 0.001$, HR 2.13, 95% CI 1.08 – 4.17, $P = 0.018$) and Glasgow (26.4 vs 16.2 months, $P = 0.011$, HR 2.37, 95% CI 0.97 – 5.79, $P = 0.048$), but not the German (22.0 vs 14.6 months, $P = 0.013$; HR 1.47, 95% CI 1.05 – 2.06, $P = 0.025$) cohort.<br>Combining S100A2 and A4 expression stratifies patient survival into 3 distinct prognostic groups in the APGI cohort (29.8 vs 17.0 vs 13.2 months, $P < 0.001$), Glasgow (26.5 vs 20.1 vs 9.3 months, $P < 0.001$) and German (22.9 vs 14.3 vs 12.9, $P < 0.001$) validation cohorts<br>Incorporating S100A2 and A4 into a prognostic pre-operative molecular nomogram predicts survival as accurately as a post-operative clinicopathological nomogram (Figure 3b, Data Supplement)<br>S100A2 and A4 can be accurately determined at pre-operative EUS biopsy (Data Supplement)
**Discussion**	S100A2 and A4 are two of the few prognostic biomarkers that have been validated in independent cohorts of patients with pancreatic cancer.<br>Their expression stratifies patients into distinct prognostic groups, and when incorporated into a pre-operative molecular nomogram, accurately predicts post-operative survival

Table S2: Availability of MSKCC prognostic variables in the validation cohorts.

MSKCC Variable	Availability APGI	Glasgow	German
Portal vein involvement	Absent	Absent	Absent
Splenectomy	Absent	Absent	Absent
Back pain	Absent	Absent	Absent
Weight loss	Absent	Absent	Absent
Posterior margin involvement	Absent	Absent	Absent
Number of nodes involved	97.7%	Absent	Absent
Number of nodes not involved	95.4%	Absent	Absent
Patient sex	Complete	Complete	Absent
Tumor longest axis length	99.2%	Complete	43.8%
Tumor location (head vs body / tail)	Complete	Complete	87.2%
Histological grade	99.6%	Complete	95.5%
Margin involvement	Complete	Complete	97.2%
T Stage	Complete	Complete	99.8%
Age at diagnosis	Complete	Complete	Complete

Table S3: Multivariate Cox Model: APGI cohort only

Variable	Coefficient	Hazard Ratio (95% CI)	***P*** Value
Differentiation (reference value: Well)			0.411
Moderate	0.287	1.33 (0.69 – 2.59)	
Poor / Undifferentiated	0.331	1.39 (0.68 – 2.83)	
Size along longest axis (cm, relative to 3.0cm)	0.050	1.05 (0.96 – 1.15)	0.013
pT Stage T3 or T4 (reference value: T1 or T2)	0.414	1.51 (0.99 – 2.30)	0.025
Age (decades, relative to 65)	0.212	1.24 (1.06 – 1.44)	0.003
Lymph nodes positive	– 0.143	0.87 (0.63 – 1.20)	0.965
Resection margin involved	0.548	1.73 (1.29 – 2.32)	< 0.001
Location tail (reference value: head)	0.279	1.32 (0.91 – 1.92)	0.052
Perineural invasion	0.081	1.08 (0.79 – 1.50)	0.210
Vascular invasion	0.248	1.28 (0.95 – 1.73)	0.049
S100A2 positive	0.275	1.32 (0.97 – 1.80)	0.017
S100A4 positive			0.018
0 – 6 months post resection	0.798	2.22 (0.84 – 5.86)	
6 – 12 months post resection	0.754	2.13 (1.08 – 4.17)	
12 – 24 months post resection	0.510	1.67 (1.00 – 2.76)	
Over 24 months post resection	0.051	1.05 (0.62 – 1.77)	

Table S4: Multivariate Cox Model: Glasgow cohort only

Variable	Coefficient	Hazard Ratio (95% CI)	***P*** Value
Differentiation (reference value: Well)			0.053
Moderate	– 0.241	0.79 (0.42 – 1.46)	
Poor / Undifferentiated	– 0.002	1.00 (0.52 – 1.93)	
Size along longest axis (cm, relative to 3.0cm)	0.366	1.44 (1.23 – 1.69)	< 0.001
pT Stage T3 or T4 (reference value: T1 or T2)	0.489	1.63 (0.93 – 2.85)	0.065
Age (decades, relative to 65)	– 0.246	1.78 (0.66 – 0.93)	0.007
Lymph nodes positive	0.164	1.18 (0.74 – 1.87)	0.020
Resection margin involved	0.560	1.80 (1.22 – 2.66)	0.022
Location head (reference value: tail)	ND*	ND*	ND*
Perineural invasion	– 0.108	0.90 (0.44 – 1.84)	0.541
Vascular invasion	0.189	1.21 (0.85 – 1.71)	0.132
S100A2 positive	0.688	2.00 (1.36 – 2.90)	< 0.001
S100A4 positive			0.048
0 – 6 months post resection	0.774	2.17 (0.74 – 6.39)	
6 – 12 months post resection	0.862	2.37 (0.97 – 5.79)	
12 – 24 months post resection	0.552	1.74 (0.94 – 3.22)	
Over 24 months post resection	– 0.152	0.86 (0.45 – 1.48)	

* All Glasgow patients had tumours in the head of the pancreas.

**Table S5:** Multivariate Cox Model: German cohort only			
Variable	Coefficient	Hazard Ratio (95% CI)	***P*** Value
Differentiation (reference value: Well)			0.099
Moderate	0.339	1.40 (0.42 – 4.73)	
Poor / Undifferentiated	0.452	1.57 (0.47 – 5.30)	
Size along longest axis (cm, relative to 3.0cm)	0.128	1.14 (0.93 – 1.39)	0.075
pT Stage T3 or T4 (reference value: T1 or T2)	– 0.208	0.81 (0.34 – 1.96)	0.973
Age (decades, relative to 65)	0.223	1.25 (1.00 – 1.56)	0.041
Lymph nodes positive	0.162	1.18 (0.76 – 1.82)	0.579
Resection margin involved	0.474	1.61 (1.08 – 2.39)	0.077
Location head (reference value: tail)	– 0.051	0.95 (0.41 – 2.25)	0.963
Perineural invasion	0.443	1.56 (1.05 – 2.30)	0.021
Vascular invasion	ND*	ND*	ND*
S100A2 positive	0.391	1.48 (0.95 – 2.29)	0.076
S100A4 positive			0.340
0 – 6 months post resection	0.904	2.47 (0.71 – 8.61)	
6 – 12 months post resection	0.262	1.30 (0.62 – 2.71)	
12 – 24 months post resection	0.091	1.10 (0.58 – 2.07)	
Over 24 months post resection	– 0.440	0.64 (0.31 – 1.32)	

* Excluded from the model to resolve collinearities.

**Table S6:** The association between S100A2 and S100A4 expression in all cohorts			
	S100A4 Negative	S100A4 Positive	***P***-value (logrank)
APGI Cohort			
S100A2 Expression			
Low	145 (88.4%)	244 (72.0%)	*P* < 0.001
High	19 (11.6%)	95 (28.0%)	
Glasgow Cohort			
S100A2 Expression			
Low	57 (93.4%)	78 (56.9%)	*P* < 0.001
High	4 (6.6%)	59 (43.1%)	
German Cohort			
S100A2 Expression			
Low	102 (80.3%)	170 (65.1%)	*P* = 0.001
High	25 (19.7%)	91 (34.9%)	

**Table S7:** The association between S100A2 and S100A4 expression and adjuvant chemotherapy			
	Adjuvant Chemotherapy	No Adjuvant Chemotherapy	***P***-value (logrank)
APGI Cohort			
S100A2 & A4 Negative	68 (46.9%)	77 (53.1%)	*P* = 0.025
S100A2 or A4 Positive	126 (47.9%)	137 (52.1%)	
Both Positive	60 (63.2%)	35 (36.8%)	
Glasgow Cohort			
S100A2 & A4 Negative	27 (47.4%)	30 (52.6%)	*P* = 0.065
S100A2 or A4 Positive	48 (58.5%)	34 (41.5%)	
Both Positive	38 (64.4%)	21 (35.6%)	
German Cohort			
S100A2 & A4 Negative	90 (70.9%)	37 (29.1%)	*P* = 0.851
S100A2 or A4 Positive	123 (62.4%)	74 (37.6%)	
Both Positive	67 (73.6%)	24 (26.4%)	

Table S8: The association between Bailey sub-type and biomarker mRNA expression in APGI cohort [RNA sequencing] (*n* = 96)

	Non-Squamous	Squamous	***P*-value (logrank)**
S100A2 mRNA expression			
Low	44 (62.0%)	4 (16.0%)	<0.001
High	27 (38.0%)	21 (84.0%)	
S100A4 mRNA expression			
Low	41 (57.7%)	7 (28.0%)	0.009
High	30 (42.3%)	18 (72.0%)	

Table S9: The association between Bailey sub-type, biomarker mRNA expression and biomarker immunohistochemistry in APGI cohort [micro-array analysis] (*n* = 235)

	Non-Squamous	Squamous	***P*-value (logrank)**
mRNA Expression			
S100A2 Expression			
Low	123 (90.4%)	41 (71.9%)	0.002
High	13 (9.6%)	16 (28.1%)	
mRNA Expression			
S100A4 Expression			
Low	52 (38.2%)	7 (12.3%)	< 0.001
High	84 (61.8%)	50 (87.7%)	
Immunohistochemistry			
S100A2 & A4 Negative	49 (36.0%)	7 (12.3%)	
S100A2 or A4 Positive	77 (56.6%)	34 (59.6%)	< 0.001
Both Positive	10 (7.4%)	16 (28.1%)	
Pre-Operative Nomogram mean score (95% CI)	103	140	0.004

Table S10: The association between tumor location and biomarker expression in APGI cohort

	Head	Body / Tail	***P*-value (logrank)**
S100A2 Expression			
Low	338 (80.9%)	54 (60.7%)	< 0.001
High	80 (19.1%)	35 (39.3%)	
S100A4 Expression			
Negative	147 (34.8%)	22 (24.2%)	0.032
Positive	276 (65.2%)	69 (75.8%)	
S100A2 & A4 Negative	130 (31.3%)	15 (17%)	
S100A2 or A4 Positive	219 (52.8%)	44 (50%)	< 0.001
Both Positive	66 (15.9%)	29 (33%)	
Pre-Operative Nomogram mean score (95% CI)	108	134	< 0.001

Table S11: S100A2 and A4 expression in paired EUS-FNA and resection specimens (PDAC denotes Pancreatic Ductal Adenocarcinoma)

	S100A2		S100A4	
Histological Diagnosis	EUS	Resection	EUS	Resection
PDAC	Low	Low	Negative	Positive
PDAC	High	High	Negative	Negative
PDAC (background of IPMN)	Low	Low	Positive	Positive
PDAC	Low	Low	Negative	Negative
PDAC	Low	Low	Positive	Positive
PDAC	High	Low	Positive	Positive
PDAC	Low	Low	Negative	Positive
PDAC	Low	Low	Negative	Negative
PDAC	Low	Low	Positive	Positive
PDAC	Low	Low	Positive	Positive
PDAC	High	Low	Negative	Negative
PDAC	Low	Low	Positive	Positive
PDAC	Low	Low	Positive	Positive
PDAC	Low	Low	Negative	Negative
PDAC	Low	Low	Negative	Positive
PDAC	Low	Low	Negative	Negative
PDAC	Low	Low	Negative	Negative

Supplementary Figures

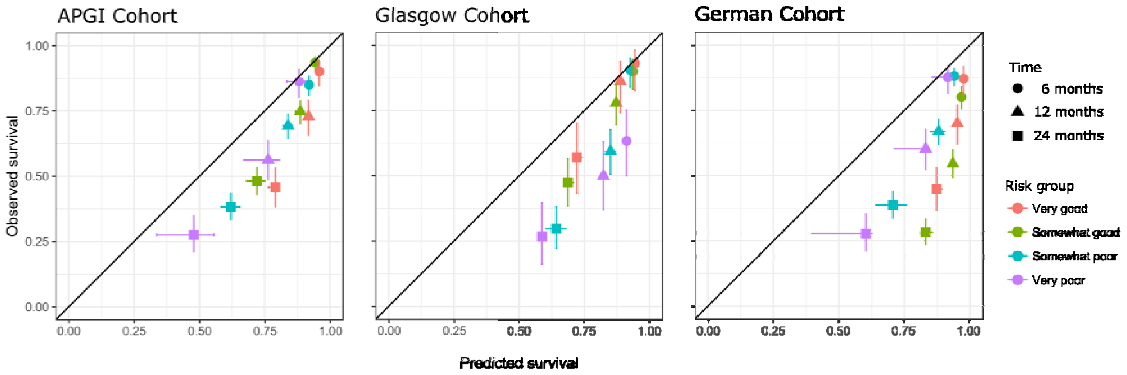

Supplementary figure 1

Supplementary figure 1: Comparison of observed and MSKCC-predicted survival in APGI, Glasgow and German cohorts. Patients were divided into four risk groups by MSKCC risk score, and the observed and nomogram-predicted survival within each risk group was compared. Error bars denote the interdecile range (for predicted survival), or the 80% binomial confidence interval (observed survival). In all three cohorts the MSKCC nomogram consistently predicts longer survival than was observed, and displays particularly poor discrimination in the German cohort.

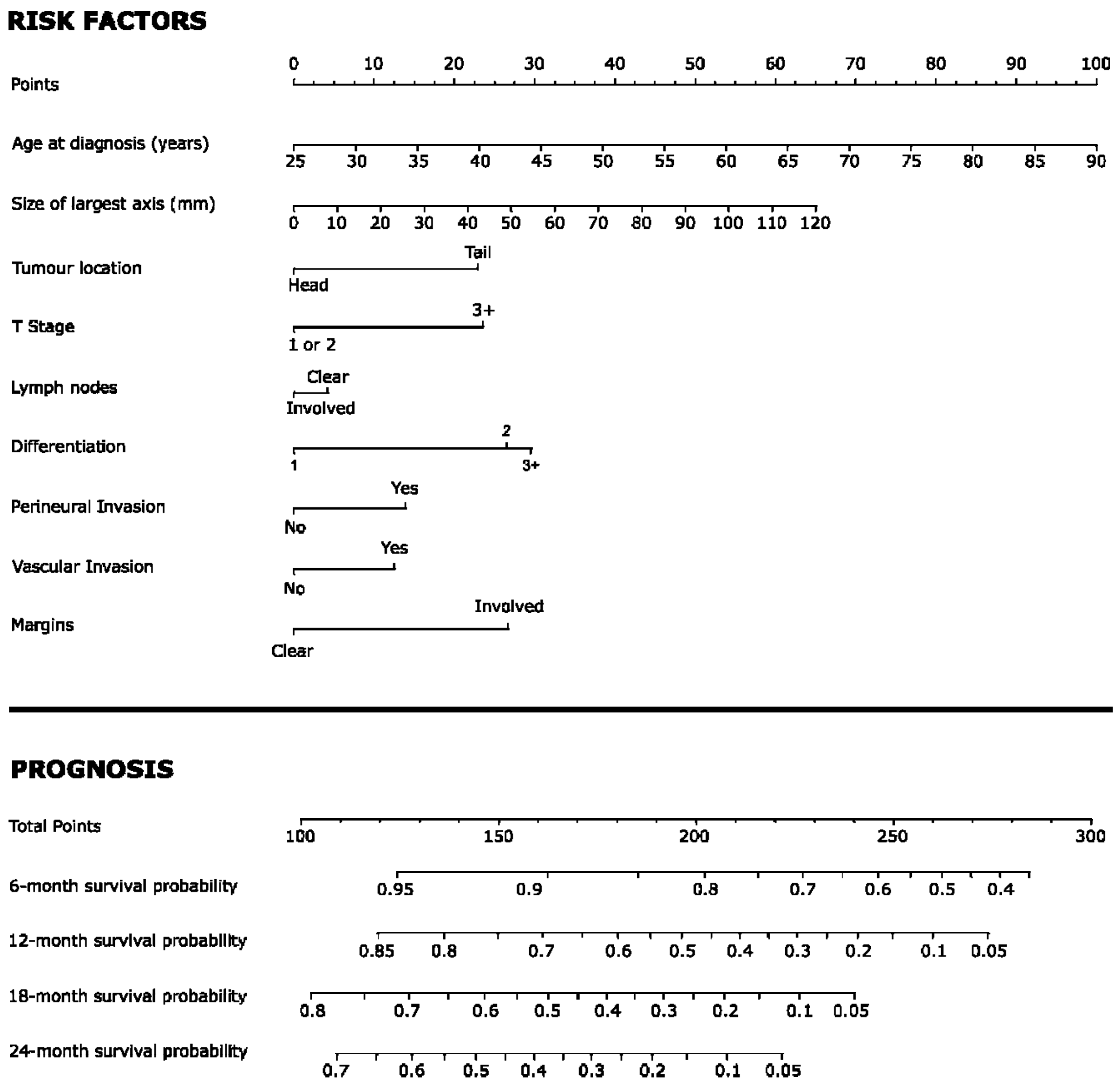

Supplementary Figure 2: Post-operative molecular prognostic nomogram for resectable pancreatic cancer.

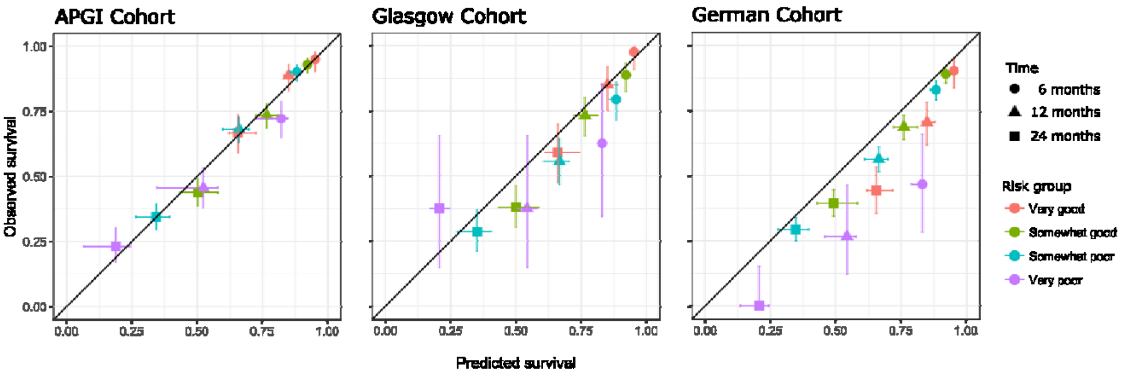

Supplementary Figure 3: Comparison of observed and APGI pre-operative nomogram predictions of survival in all cohorts. Patients were divided into four risk groups by APGI preoperative risk score, and the observed and nomogram-predicted survival within each risk group was compared. Error bars denote the interdecile range (for predicted survival), or the 80% binomial confidence interval (observed survival).

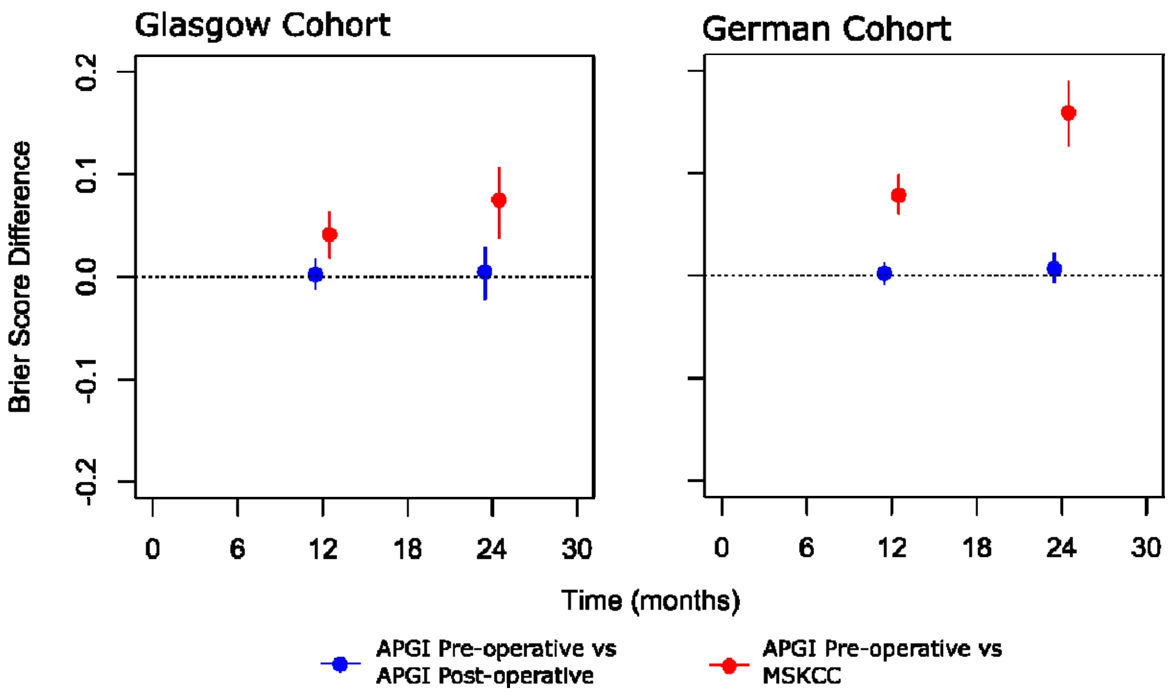

Supplementary Figure 4: Comparison of the validation cohorts' overall prediction accuracy between APGI pre-operative, APGI post-operative, and MSKCC post-operative nomograms. Differences in Brier score between the APGI pre-operative, and either the APGI or MSKCC post-operative nomograms, are shown at two time points (12 & 24 months). Positive values indicate lower error in the APGI pre-operative nomogram compared to the APGI post-operative and MSKCC nomograms. Points denote modal values, and bars denote 90% highest posterior density intervals, over 5,000 bootstrap resamples of each validation cohort.

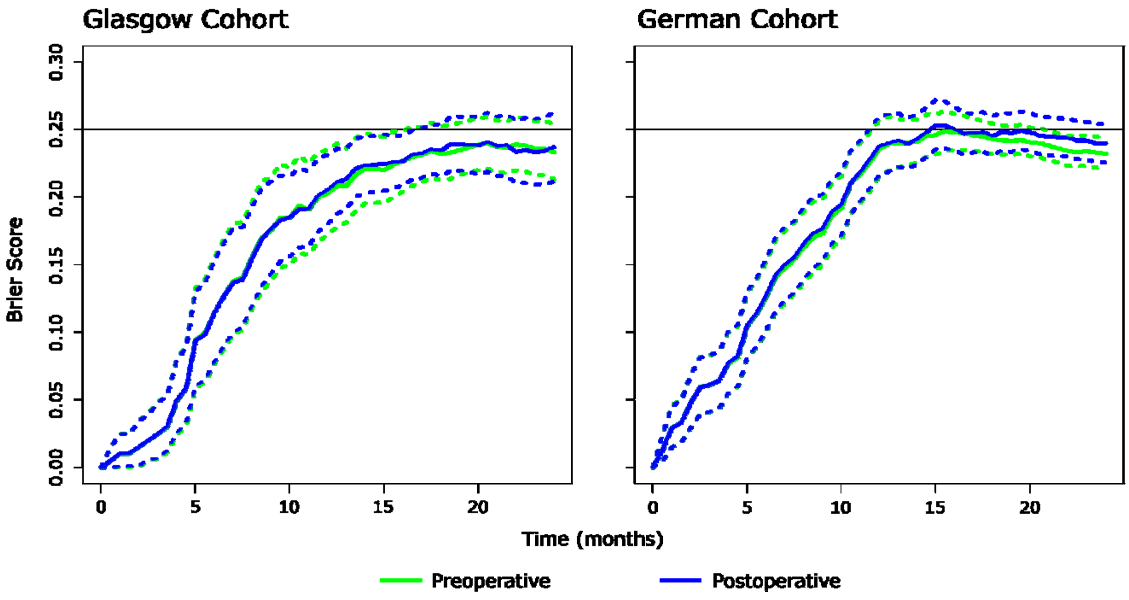

Supplementary Figure 5: Brier score plots demonstrate the APGI pre-operative nomogram predicts prognosis as accurately as the APGI post-operative nomogram in the Glasgow and German cohorts

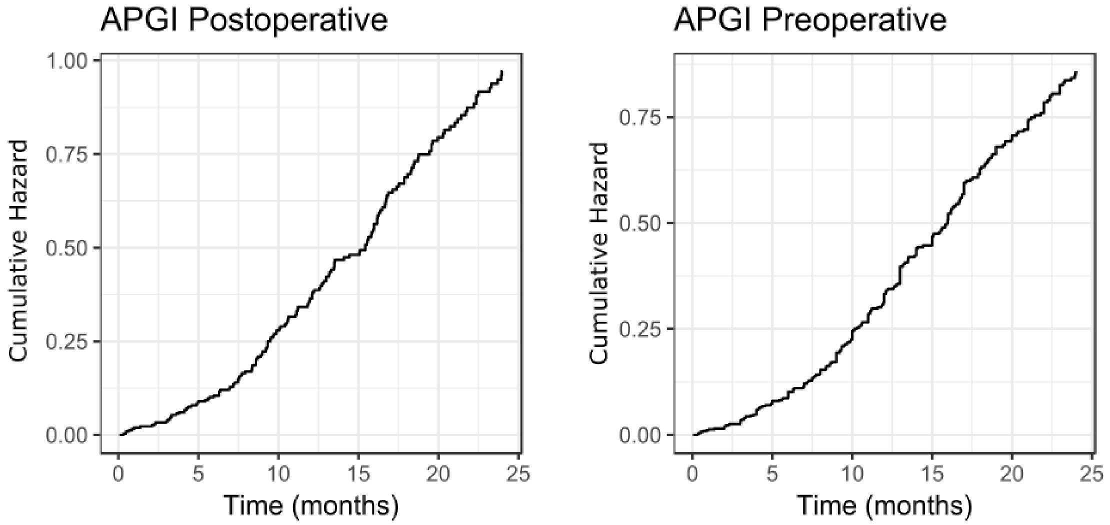

Supplementary Figure 6: Baseline hazards (expressed as cumulative hazard) for the APGI-trained preoperative and postoperative nomograms.

References

1. Hudson TJ, Anderson W, Artez A, et al: International network of cancer genome projects. Nature 464:993-8, 2010

2. Biankin AV, Waddell N, Kassahn KS, et al: Pancreatic cancer genomes reveal aberrations in axon guidance pathway genes. Nature 491:399-405, 2012

3. Waddell N, Pajic M, Patch AM, et al: Whole genomes redefine the mutational landscape of pancreatic cancer. Nature 518:495-501, 2015

4. Jamieson NB, Mohamed M, Oien KA, et al: The relationship between tumor inflammatory cell infiltrate and outcome in patients with pancreatic ductal adenocarcinoma. Ann Surg Oncol 19:3581-90, 2012

5. Bailey P, Chang DK, Nones K, et al: Genomic analyses identify molecular subtypes of pancreatic cancer. Nature 531:47-52, 2016

6. Mann KM, Ward JM, Yew CC, et al: Sleeping Beauty mutagenesis reveals cooperating mutations and pathways in pancreatic adenocarcinoma. Proc Natl Acad Sci U S A 109:5934-41, 2012

7. Chang DK, Johns AL, Merrett ND, et al: Margin clearance and outcome in resected pancreatic cancer. J Clin Oncol 27:2855-62, 2009

8. Murphy NC, Scarlett CJ, Kench JG, et al: Expression of LMO4 and outcome in pancreatic ductal adenocarcinoma. Br J Cancer 98:537-41, 2008

9. Biankin AV, Kench JG, Colvin EK, et al: Expression of S100A2 calcium-binding protein predicts response to pancreatectomy for pancreatic cancer. Gastroenterology 137:558-68, 568.e1-11, 2009

10. Collins GS, Reitsma JB, Altman DG, et al: Transparent Reporting of a multivariable prediction model for Individual Prognosis Or Diagnosis (TRIPOD). Ann Intern Med 162:735-6, 2015

11. Neoptolemos JP, Stocken DD, Bassi C, et al: Adjuvant chemotherapy with fluorouracil plus folinic acid vs gemcitabine following pancreatic cancer resection: a randomized controlled trial. JAMA 304:1073-81, 2010

12. Schnelldorfer T, Ware AL, Sarr MG, et al: Long-term survival after pancreatoduodenectomy for pancreatic adenocarcinoma: is cure possible? Ann Surg 247:456-62, 2008

13. Cameron JL, Riall TS, Coleman J, et al: One thousand consecutive pancreaticoduodenectomies. Ann Surg 244:10-5, 2006

14. Biankin AV, Kench JG, Colvin EK, et al: Expression of S100A2 calcium-binding protein predicts response to pancreatectomy for pancreatic cancer. Gastroenterology 137:558-68, 2009

15. Bland JM, Altman DG: The logrank test. BMJ 328:1073, 2004

16. Groot VP, Rezaee N, Wu W, et al: Patterns, Timing, and Predictors of Recurrence Following Pancreatectomy for Pancreatic Ductal Adenocarcinoma. Ann Surg, 2017
